# Diagnosis and management of traumatically induced hemicrania continua and neuropathic pain secondary to facial gun shot wound

**DOI:** 10.1111/joor.13324

**Published:** 2022-04-11

**Authors:** Gary M. Heir, Louis DiPede, Manvitha Kuchukulla, Mythili Kalladka, Shahad Aziz

**Affiliations:** ^1^ Program Director, Center for Temporomandibular Disorders and Orofacial Pain Rutgers School of Dental medicine Newark New Jersey USA; ^2^ Restorative Dentistry Kornberg School of Dentistry Temple University Philadelphia Pennsylvania USA; ^3^ 212493 Post‐Graduate Program in Orofacial Pain Center for Temporomandibular Disorders and Orofacial Pain Rutgers School of Dental Medicine Newark New Jersey USA; ^4^ Orofacial Pain and TMD Eastman Institute of Oral Health Rochester New York USA; ^5^ 212493 Department of Oral and Maxillofacial Surgery Rutgers School of Dental Medicine Newark New Jersey USA

**Keywords:** cephalgia, Horner's, oralsurgery, pharmacotherapy, prosthodontics, trigeminal

## Abstract

Near fatal gunshot wound to the face results in lifesaving surgery and restorative procedures. Chronic pain followed. This is the probable first case report of posttraumatic hemicrania continua and its successful management.

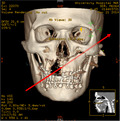

## 1 INTRODUCTION

**FIGURE 1:**
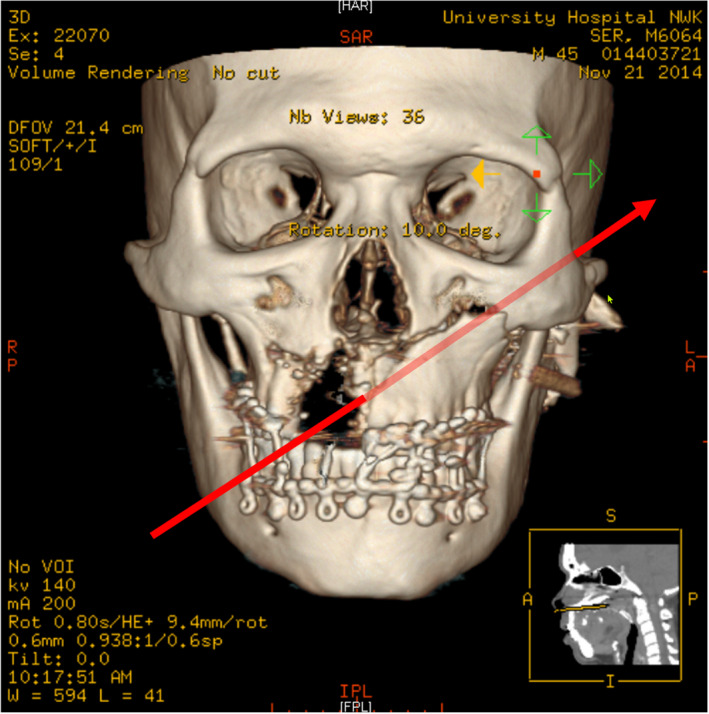
A‐P CBCT view of the entry wound. Note bullet trajectory and potential for trigeminal and facial nerve at *the site of the exit wound*

After life‐saving measures following a gunshot in the face, a 53‐year‐old man underwent several surgical procedures to repair the palate and left temporomandibular joint (TMJ). The patient was initially evaluated status post‐gunshot wound (Figure 1). He sustained multiple facial fractures including a comminuted maxillary component to the gunshot wound resulting in a large palatal defect. Based on the size of the defect and the nature of the surrounding soft tissue, this large fistula was treated with a local pedicle flap. This required a 2‐stage procedure with a tongue pedicle for approximately 2–3 weeks, followed by its release under generalised anaesthesia.

**FIGURE 2:**
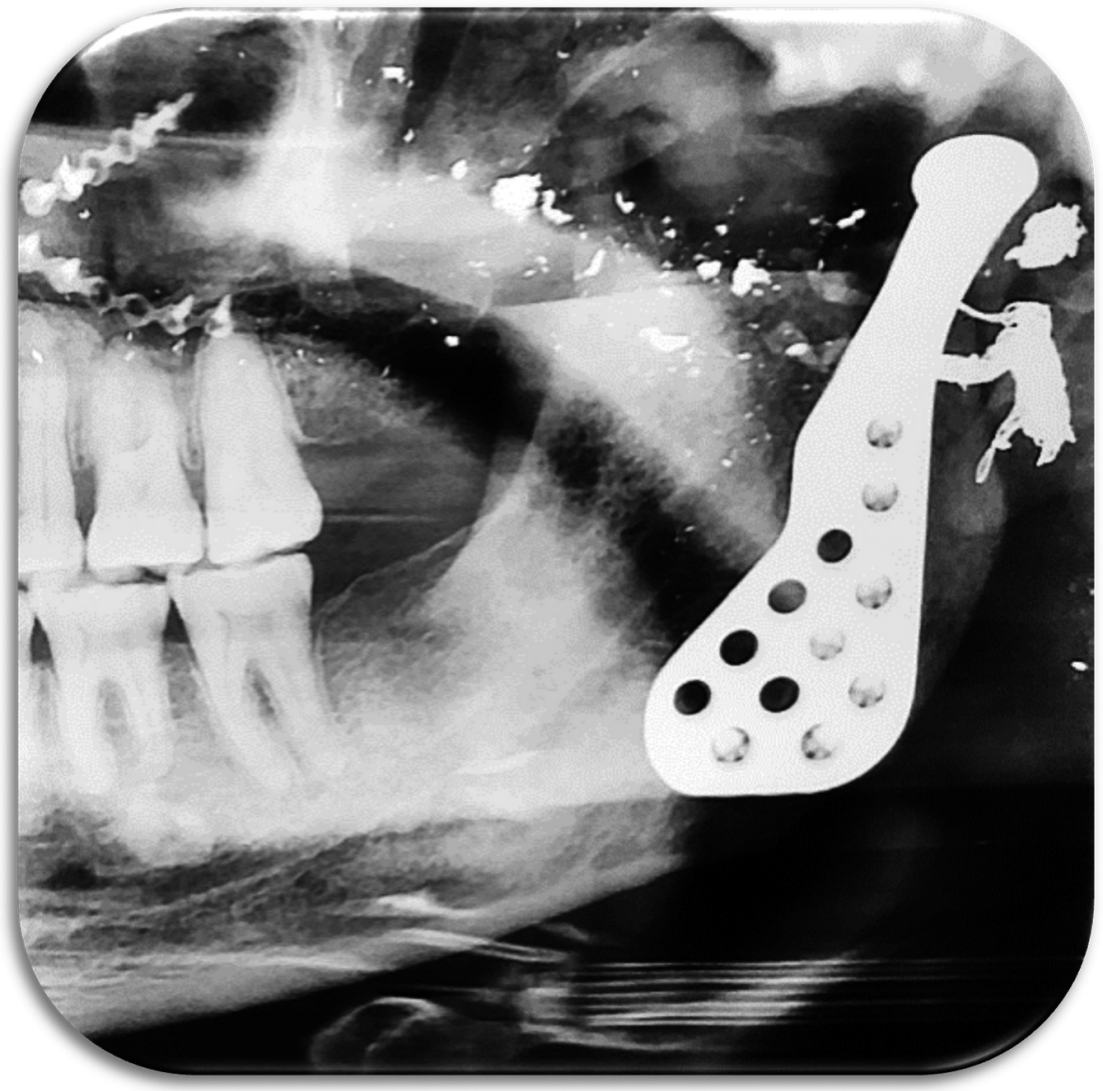
The left temporomandibular prosthesis is in place. The scatter of shrapnel throughout the area

At approximately one year following the incident, the patient underwent an open reduction of a comminuted condylar fracture in an attempt to salvage the condyle. However, he developed progressive trismus and subsequent ankylosis of the left TMJ limiting the mandibular range of motion. Reconstruction of the left TMJ was required to restore mandibular function. A Biorret prosthetic joint replaced the destroyed left mandibular condyle (Figure 2).^1^ Following surgical procedures, including placement of a prosthetic left TMJ, the patient was referred for rehabilitation of the mandibular range of motion in order to proceed with prosthodontic reconstruction.

The post‐surgical mandibular opening was limited to 5 mm, making prosthodontic treatment impossible.

The mandibular range of motion was adequately restored following the left total prosthetic joint replacement using physical therapy modalities including progressive mandibular depression facilitated by moist and radiant heat. This effectively restored the mandibular range of movement to approximately 28 mm, more than adequate to provide the necessary prosthodontics restorations.

During the initial evaluation for the orthopaedic rehabilitation phase of treatment, clinical findings revealed additional signs and symptoms consistent with left‐sided headache associated with unilateral autonomic features, neuropathic pain in the zygomatic distribution of CN‐V_2_, and paralysis of the left forehead consistent with palsy of the temporal branch of the facial nerve CN‐VII. None of these complaints were present prior to the assault and subsequent surgeries, and went unreported by the patient until the comprehensive orofacial pain evaluation.

## 2 CLINICAL EVALUATION

The orofacial pain evaluation included a detailed history, comprehensive clinical evaluation, judicious use of adjunctive diagnostic testing, formulation of a differential diagnosis and implementation of a treatment plan. The minimal information necessary for an adequate history includes, but is not limited to the chief complaint, a review of systems, past medical history, review of the location, duration, frequency, intensity, precipitating and ameliorating factors related to any pain complaint, current and past medications or treatments and efficacy of past treatment. The evaluation began with clinical observation.

**FIGURE 3:**
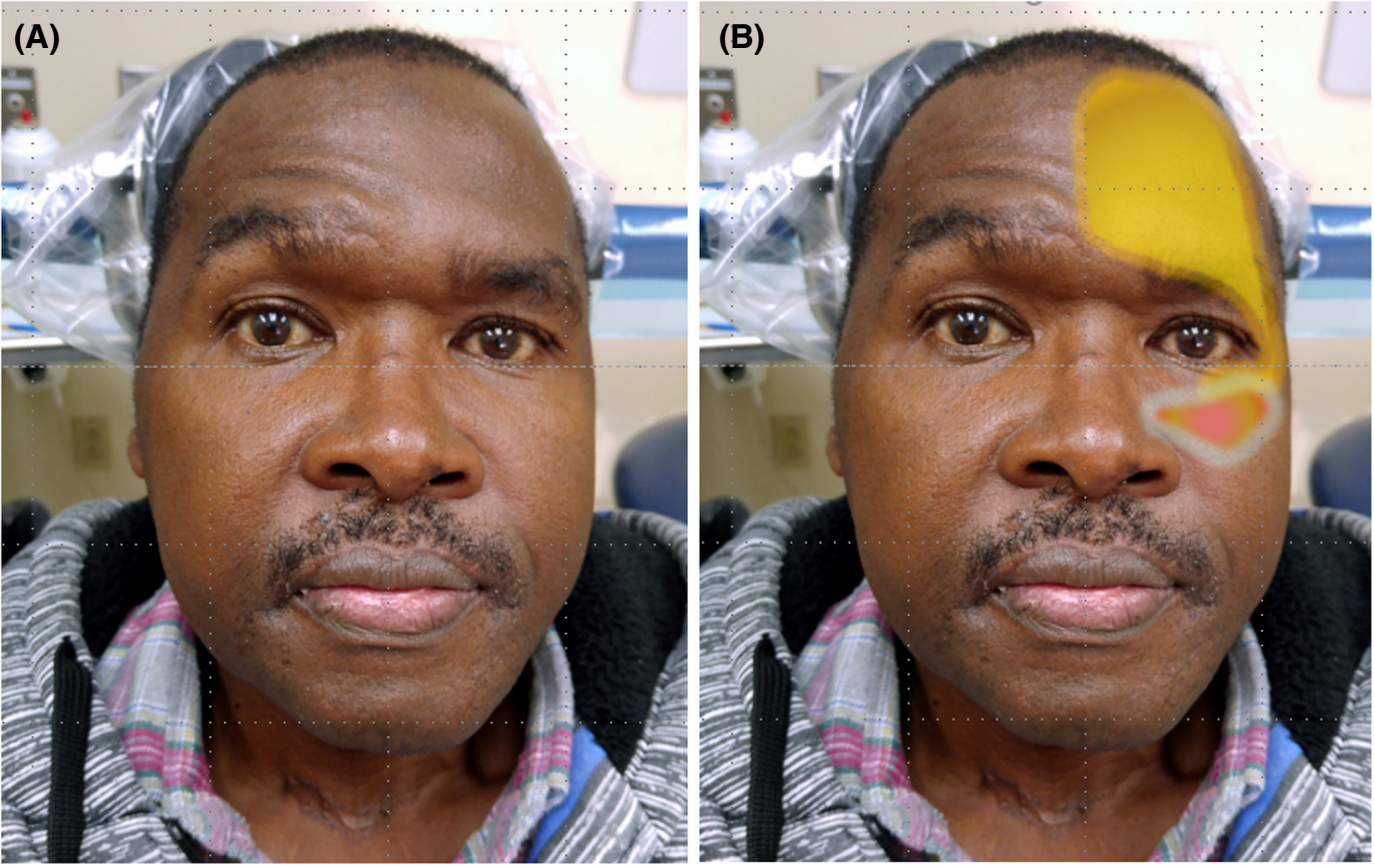
(A) (left) The patient's appearance upon presentation at the initial evaluation. (B) (right) The area highlighted in yellow designates continuous pain associated with autonomic features, the area in red designates continuous burning and tingling pain associated with allodynia and hyperalgesia (*The patient has given written authorisation for the use of his unredacted photos*)

In this case, the chief complaint of limited mandibular range of movement was obvious. However, careful observation found much more. In addition to the limited mandibular function and pain of the masticatory muscles, the patient reported a second complaint of headache with periorbital pain and a burning and tingling sensation along the left zygomatic arch. The left forehead was flat compared to the right with the left eyebrow much lower than on the right. The left eye appeared smaller than the right, and the eyelid was puffy, consistent with ptosis (Figure 3A,B). Anisocoria was present with asymmetrical pupillary size; the left was somewhat smaller than the right. The left sclera was reddened with an injected appearance. During the interview, the patient was observed constantly wiping his left eye and left nostril, due to persistent rhinorrhoea and lacrimation. He was also observed turning away from the windows, shielding his left eye from the light. He described photophobia, explaining that he was ‘coming down from an attack’. These autonomic features associated with left‐sided headache were consistent with Horner's Syndrome. Headache was constant with background discomfort waxing and waning throughout the day from levels of moderate discomfort, escalating to severe episodes that might occur at any time. Background discomfort was constant with occasional ‘attacks’, exacerbations as described above. It was during these ‘attacks’ the patient experienced the associated autonomic features of lacrimation and rhinorrhoea. He said that since being shot, ‘this has never left’. He denied prior symptoms of a similar nature and assumed that these symptoms were the residual effects of the trauma and nothing could be done.

An additional complaint was of a burning and tingling sensation extraorally, across the left zygomatic arch. A cranial nerve screening evaluation revealed allodynia, a painful response to a non‐noxious stimulus, and hyperalgesia, a greater response to a noxious stimulus than that would be anticipated, in the distribution of the zygomatic branch of CN‐V_2_. Discomfort in this area was affected by stress and cold weather, associated with intensification of symptoms. This area, supplied by the second division of the trigeminal nerve, CN‐V_2_, was likely traumatised by the trajectory of the bullet and the resultant tissue damage. This second condition was diagnosed as painful post‐traumatic trigeminal neuropathy (PPTTN). This condition secondary to trauma to a branch of the trigeminal nerve resulting in persistent pain with sensory changes.

### 2.1 Summary of chief complaints

- Limited mandibular opening and masticatory musculoskeletal pain.
- Left half‐head headache and periorbital pain associated with autonomic features.
- Facial nerve paralysis involving the forehead.
- Burning and tingling pain of the left zygomatic region.

## 3 DIFFERENTIAL DIAGNOSES

Cluster headache (CH), chronic paroxysmal hemicrania (CPH) and HC often mimic anterior maxillary odontogenic pain leading to inappropriate dental procedures. Therefore, a differential diagnosis must also include odontalgia. Differential diagnoses also considered in this, and similar cases are temporomandibular disorders and trigeminal neuralgia. With the elimination of all other potential diagnoses and consistent with the clinical presentation. The diagnoses included:

- Post‐traumatic hemicrania continua.
- Post‐traumatic trigeminal neuropathy of CN‐V_2_.
- Palsy of the temporal branch of the left facial nerve secondary to trauma.
- Limited mandibular range of motion with masticatory muscle pain.

Diagnostic considerations could have included worsening of pre‐existing migraine or tension‐type headaches following trauma.^2^ However, there was no history of prior migraine or neurovascular orofacial pain prior to the assault and subsequent surgeries. Symptoms that evolved following the gunshot wound and subsequent surgeries went unreported.

## 4 PATHOPHYSIOLOGY AND AETIOLOGY

### 4.1 Hemicrania continua

Trigeminal autonomic cephalgias (TACs) are a group of primary idiopathic headache disorders. TACs include chronic hemicranias (CH), chronic paroxysmal hemicranias (CPH), hemicranias continua (HC) short‐lasting unilateral, neuralgiform headache with conjunctival injection (SUNCT), and short‐lasting, unilateral neuralgiform headache attacks with cranial autonomic features (SUNA; Table 1). Post‐traumatic TAC is reported following a head injury.^3^ The suggested mechanism for post‐traumatic migraine is secondary to axonal injury or a shearing effect of the brainstem, even after mild or moderate injury. A physiological shift within the brainstem can lead to chronic migraine.^4^ Similar post‐traumatic physiological events are associated with the onset of SUNCT or SUNA, suggesting an abnormality in the brainstem or hypothalamus, or the activation of a pre‐existing predisposition to such abnormality.^5^ Another potential mechanism is the activation of trigeminovascular nociceptive pathways, which activates a trigemino‐autonomic reflex resulting in headaches.^6^

**TABLE 1:** Trigeminal autonomic cephalalgias (TACs) – summary of differentiating features of various TACs^19^, ^20^

	Cluster headache	Paroxysmal hemicrania	Hemicrania continua	SUNCT/SUNA
Age/gender (M:F) predilection	20–40 years 5:1	30–40 years 1:1.6		50 years 1.5:1
Location	Orbital/supraorbital and temporal region	Temporal, periorbital, orbital, maxillary areas	Frontal, temporal and periorbital region	Periorbital
Duration	15–180 min	2–30 min	Continuous with periods of exacerbation	5–240 s
Frequency of attacks	1 attack every alternate day Up to 8/day	Up to 40/day	Continuous	3–200/day
Characteristics of pain	Stabbing/sharp/boring/piercing/stabbing	Throbbing/stabbing/sharp/boring	Throbbing/feeling of foreign body/sand in the eye	Excruciating, stabbing
Intensity	Severe	Severe	Moderate‐severe	Severe
Aggravating factors	Alcohol, histamine, nitro‐glycerine		Menses, stress, bending over, strong odours	Light touch may precipitate attack
Autonomic features	Present	Present	Present	Present
Associated features	Migrainous features, prodromal or premonitory symptoms and occasionally hemiparesis may be present, Patient may be restless and agitated	Migrainous features may be present	Migrainous features may be present	Cutaneous trigger zones may be present
Response to Indomethacin	No	Yes	Yes	No
Treatment	Abortive‐Oxygen inhaled through face mask, triptan, dihydroergotamine, Prophylactic‐Verapamil, Prednisone	Indomethacin	Indomethacin	Lamotrigine, gabapentin

TACs are unilateral, considered side‐locked, and associated with autonomic features (Table 2). TACs have common features, with the exception of HC, in that they are typically episodic; HC is an uncommon, benign unilateral and continuous headache, with superimposed exacerbations of severe pain and autonomic features (Table 3). Other features may include photophobia, phonophobia or both, nausea and ocular discomfort.

**TABLE 2:** IHS classification ICHD‐3 trigeminal autonomic cephalgia^22^

3. Trigeminal autonomic cephalalgias (TACs)
3.1 Cluster headache
3.1.1 Episodic cluster headache
3.1.2 Chronic cluster headache
3.2 Paroxysmal hemicrania
3.2.1 Episodic paroxysmal hemicrania
3.2.2 Chronic paroxysmal hemicrania
3.3 Short‐lasting unilateral neuralgiform headache attacks
3.3.1 Short‐lasting unilateral neuralgiform headache attacks with conjunctival injection and tearing (SUNCT)
3.3.1.1 Episodic SUNCT
3.3.1.2 Chronic SUNCT
3.3.2 Short‐lasting unilateral neuralgiform headache attacks with cranial autonomic symptoms (SUNA)
3.3.2.1 Episodic SUNA
3.3.2.2 Chronic SUNA
3.4 Hemicrania continua
3.4.1 Hemicrania continua, remitting subtype
3.4.2 Hemicrania continua, unremitting subtype
3.5 Probable trigeminal autonomic cephalgia
3.5.1 Probable cluster headache
3.5.2 Probable paroxysmal hemicrania
3.5.3 Probable short‐lasting unilateral neuralgiform headache attacks
3.5.4 Probable hemicrania continua

**TABLE 3:** Diagnostic criteria, hemicrania continua^22^

Unilateral headache fulfilling criteria B–D
Present for >3 months, with exacerbations of moderate or greater intensity
Either or both of the following:
At least one of the following symptoms or signs, ipsilateral to the headache:
– Conjunctival injection and/or lacrimation
– Nasal congestion and/or rhinorrhea
– Eyelid oedema
– Forehead and facial sweating
– >Miosis and/or ptosis
A sense of restlessness or agitation, or aggravation of the pain by movement
Responds absolutely to therapeutic doses of indomethacin1
Not better accounted for by another ICHD‐3 diagnosis
Note:
In an adult, oral indomethacin should be used initially in a dose of at least 150 mg daily and increased if necessary up to 225 mg daily. The dose by injection is 100–200 mg. Smaller maintenance doses are often employed
Comments:
Migrainous symptoms such as photophobia and phonophobia are often seen in 3.4 Hemicrania continua
3.4 Hemicrania continua is included under 3. Trigeminal autonomic cephalalgias in ICHD‐3 on the basis that the pain is typically unilateral, as are the cranial autonomic symptoms when present (in ICHD‐II it was under 4. Other primary headache disorders)
Brain imaging studies show important overlaps between all disorders included here, notably activation in the region of the posterior hypothalamic grey. In addition, the absolute response to indomethacin of 3.4 Hemicrania continua is shared with 3.2 Paroxysmal hemicrania

The pathophysiology of TAC is not completely elucidated. A combination of both central and peripheral mechanisms may be involved in the genesis of TACs with a predominant central component. Proposed theories of TAC include trigeminovascular system activation,^7^ sympathetic dysfunction, hypothalamic dysfunction, perivascular neurogenic inflammation of the internal carotid artery (ICA) and the role of subcortical neural structures.

Activation of the trigeminovascular system or the trigeminal nerve may lead to autonomic symptoms in some patients. Autonomic symptoms of TAC may also be due to sympathetic dysfunction. Sympathetic dysfunction may be generalised or secondary to effects of carotid oedema on the associated sympathetic plexus resulting in effects similar to neuropraxia.^8^ Previous studies have shown that peripheral neuroinflammation/neuritis may induce signs of neuropraxia and induce changes in corresponding sensory ganglia and the central nervous system.^9^ Alternately, stimulation of CN‐V_1_ and CN‐V_2_ of the trigeminal nerve by noxious stimuli induces symptoms similar to autonomic symptoms of TAC through activation of the trigemino‐parasympathetic reflex.^10^ Another proposed mechanism for TAC is hypothalamic dysfunction. The trigeminal nuclei and pterygopalatine ganglion have functional connections with the hypothalamus. The hypothalamus plays an important role in pain processing and modulates the trigemino‐parasympathetic reflex.^11^, ^12^

While the pathophysiology of traumatic TAC, especially HC, is poorly understood, there are instances where the onset of a TAC is associated with trauma as seems to be the case here. Headaches, most commonly migraine, tension‐type headaches and rarely CH are reported following head injury^13^; however, case reports of traumatic HC are extremely rare.^14^ It is suggested that central, peripheral mechanisms and a combination of central and peripheral mechanisms may be involved in the pathogenesis of traumatic TAC such as CH and CPH.

TACs may arise as a direct result of trauma, noting the possibility of risk factors in genetically predisposed individuals. One recent study reported that chronic CH was more frequently associated with a positive family history and a history of traumatic head injury.^15^ A cohort study on headache reported by military personnel with a history of traumatic brain injury reported the most common form was due to blast injuries, and 7.2% of the subjects were diagnosed with HC.^16^ It is assumed that peripheral changes in the trigeminal pathway following trauma, including sensitisation of peripheral nociceptors, neuroinflammation and mechanical damage, may modulate structural changes and reorganisation in the central nervous system (CNS) as another mechanisms for post‐traumatic TAC/CH, especially in a predisposed patient.^13^

Several forms of TACs, including HC, are sensitive to indomethacin and referred to as indomethacin responsive headaches (IRH). HC is dramatically responsive to indomethacin, classifying it as an IRH. A positive response to indomethacin, ranging from 25 to 300 mg daily, is considered diagnostic.^17^ The specific pharmacology of indomethacin in headache therapy unknown. The hypothesis is, indomethacin acts as a cyclooxygenase inhibitor, has interactions with cell signalling and inflammatory pathways, and may have an effect on nitric oxide synthesis resulting in its antinociceptive action.^18^

The flat appearance of the ipsilateral forehead and lowered eyebrow is consistent with facial nerve trauma but did not explain ptosis. Ptosis, including limited elevation of the eyelid seen in Horner's syndrome associated with TACs, is mediated by the oculomotor or third cranial nerve (CN‐III), not CN‐V or CN‐VII. This phenomenon required explanation. The trajectory of the project could not have involved the oculomotor nerve.

A single case series of traumatic HC has been reported.^14^ However, the authors do not discuss the pathogenesis of traumatic HC. It was hypothesised that comminuted fractures of the maxilla may have resulted in HC with the following possible contributory pathophysiologic mechanisms or a combination of these mechanisms:

- activation of the CN‐V2 trigeminovascular system and disinhibition of trigeminal autonomic reflex with possible structural reorganisation in the CNS,
- neuroinflammation and neuropraxic effects on the sphenopalatine ganglion,
- considering the severity of the trauma, it is also plausible that there was additional activation of the hypothalamus through its connection with the trigeminal nuclei and trigemino‐parasympathetic reflex.^21^

## 5 PAINFUL POST‐TRAUMATIC TRIGEMINAL NEUROPATHY

Painful Post‐Traumatic Trigeminal Neuropathy (PPTTN) is a condition associated with sensory changes and/or pain along the course of the innervation of a traumatised branch of the trigeminal nerve. Symptoms must be present for more than three months.^11^, ^12^

## 6 MANAGEMENT

The HC was effectively treated with indomethacin. The patient was titrated from 25 mg per day up to 75 mg per day in divided doses. After only several days he realised immediate relief from the HC and was titrated downward to the minimum effective dose of 25 mg daily.

**FIGURE 4:**
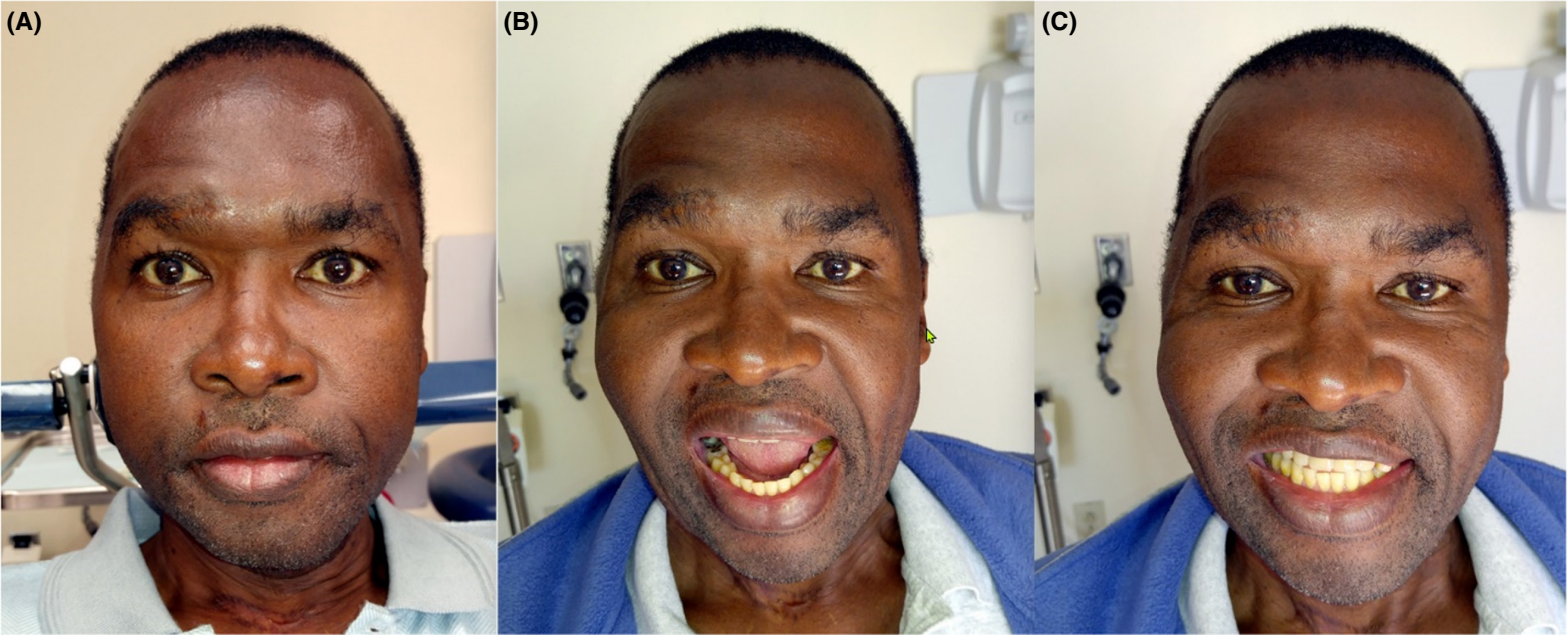
(A) The patient 10 days after commencement of pharmacotherapy including indomethacin 25 mg per day for the HC and 10 mg of nortriptyline. Note the absence of ptosis in the near equal levels of the eyebrows. Scleral injection, rhinorrhoea and lacrimation have been eliminated. The left forehead remains flat. (B and C) The patient is prosthodontically restored and pain‐free. This mandibular range of movement is more than adequate for masticatory function and speech. (The patient has given written authorisation for the use of his unredacted photos.)

Nortriptyline was prescribed, 10 mg daily at bedtime. He realised the remarkable and rapid diminution of PPTTN within one week (Figure 4A–C).

He remained completely pain‐free for two years at which point he felt breakthrough tingling in the area of the left zygomatic branch of CN‐V_2,_ low‐dose pregabalin, 75 mg three times daily, was added three years ago. The patient has been followed for more than six years and remains pain‐free on this regimen. No treatment for the facial nerve injury was possible and considered permanent.

The mandibular range of motion was rehabilitated to 28 mm with physical therapy modalities including progressive gentle stretching home care exercises with hot and cold packs.

No treatment was possible for the facial nerve injury.

The patient is followed three times annually and routine serology is collected.

## 7 CONCLUSION

This individual incurred a gunshot wound to the face involving severe trauma to the palate and left TMJ. Urgent, life‐saving efforts and surgery led to prosthetic reconstruction of his left TMJ. Prosthodontic restoration to restore masticatory function was the next most appropriate step in rehabilitation. While he was referred for management of a significantly limited mandibular range of motion, other quality of life issues became apparent during the initial evaluation. These included a TAC, neuropathic pain and facial nerve palsy. The patient was convinced these were permanent conditions, which could not be treated. A comprehensive orofacial pain evaluation led to the various diagnoses illustrated in this case report, which when identified and treated individually, resulted in significant, if not complete pain relief and improved quality of life. He is monitored 3 to 4 times per year and serology is performed biannually in cooperation with his primary care physician. The authors believe this is the first, if not one of only few case reports of post‐traumatic HC.

## CONFLICT OF INTEREST

The authors claim no conflict of interest or relationship, financial or otherwise that might be perceived as influencing an author's objectivity.

## AUTHOR CONTRIBUTIONS

Gary M. Heir: Primary author and treating clinician. Louis DiPede: Secondary author and treating prosthodontist. Manvitha Kuchukulla: Background research and editing. Mythili Kalladka: Background research, literature review, compilation of tables and editing. Shahad Aziz: Secondary author and treating oral surgeon.

## ETHICS APPROVAL

Patient consent for this publication has been given.

### PEER REVIEW

The peer review history for this article is available at https://publons.com/publon/10.1111/joor.13324.

## Data Availability

As this is a case report, the manuscript does not contain any data to be shared.
